# Foliar fungal endophyte community structure is independent of phylogenetic relatedness in an Asteraceae common garden

**DOI:** 10.1002/ece3.6983

**Published:** 2020-11-26

**Authors:** Briana K. Whitaker, Natalie Christian, Qing Chai, Keith Clay

**Affiliations:** ^1^ Department of Biology Indiana University Bloomington IN USA; ^2^ Department of Biology University of Louisville Louisville KY USA; ^3^ School of Pastoral Agriculture Science and Technology Lanzhou University Lanzhou China; ^4^ Department of Ecology and Evolutionary Biology Tulane University New Orleans LA USA

**Keywords:** biodiversity, composites, fungal endophytes, horizontal transmission, host specificity, Illumina sequencing

## Abstract

Phylogenetic distance among host species represents a proxy for host traits that act as biotic filters to shape host‐associated microbiome community structure. However, teasing apart potential biotic assembly mechanisms, such as host specificity or local species interactions, from abiotic factors, such as environmental specificity or dispersal barriers, in hyperdiverse, horizontally transmitted microbiomes remains a challenge. In this study, we tested whether host phylogenetic relatedness among 18 native Asteraceae plant species and spatial distance between replicated plots in a common garden affects foliar fungal endophyte (FFE) community structure. We found that FFE community structure varied significantly among host species, as well as host tribes, but not among host subfamilies. However, FFE community dissimilarity between host individuals was not significantly correlated with phylogenetic distance between host species. There was a significant effect of spatial distance among host individuals on FFE community dissimilarity within the common garden. The significant differences in FFE community structure among host species, but lack of a significant host phylogenetic effect, suggest functional differences among host species not accounted for by host phylogenetic distance, such as metabolic traits or phenology, may drive FFE community dissimilarity. Overall, our results indicate that host species identity and the spatial distance between plants can determine the similarity of their microbiomes, even across a single experimental field, but that host phylogeny is not closely tied to FFE community divergence in native Asteraceae.

## Graphical abstract

We tested whether foliar fungal endophyte communities are structured by host phylogenetic relatedness using a common garden experimental design and an explicit selection of host species from a defined phylogenetic range. We found that host species identity was a significant driver of foliar fungal endophyte community structure. However, we did not find that pairwise phylogenetic distance between host species was significantly correlated with foliar fungal endophyte community dissimilarity between host individuals.

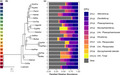

## 1 INTRODUCTION

Microorganisms have been intimately associated with macro‐organisms for hundreds of millions of years (Krings et al., 2007; Ley et al., 2008). Research across plant and animal systems, combined with the advent of advanced sequencing technologies (Alivisatos et al., 2015), has revealed a wide range of taxonomic and functional diversity in their microbiomes (Christian et al., 2015). Despite the persistence and longevity of these symbioses over evolutionary timescales, the fact that most microbiota colonize hosts via horizontal transmission from their local environment raises the question of whether environmentally acquired microbiota reflect the phylogenetic relatedness of their hosts (Christian et al., 2017) as in many pathogen (Gilbert & Webb, 2007) and mutualistic systems (Hammerstein & Noë, 2016), or whether they simply reflect spatial structuring and environmental specificity.

Host‐associated microbiomes are assembled and maintained via multiple mechanisms that act across a wide range of spatial and temporal scales. At the local level, bacterial and fungal symbionts may compete for common resources or to gain entry and occupy a particular spatial niche within the host (Hooper et al., 2012). Host phenotypic traits (e.g., morphology, physiology, tissue chemistry) can also influence microbial colonization (Laforest‐Lapointe et al., 2017). At larger spatial scales, dispersal limitation can constrain which microbial community members are able to colonize hosts and can lead to patterns of spatial distance decay in community similarity across the landscape (Oono et al., 2017). Microbial preferences for particular environmental or climatic conditions can also be an important driver of microbial community composition (Giauque & Hawkes, 2013). Likewise, the frequency and timing of disturbances can affect the ability of microbial communities to rebound to their previous composition (Shen et al., 2018).

Because the richness, diversity, and structure of horizontally transmitted microbial communities are shaped by mechanisms acting across such a wide range of spatial and temporal scales, it has been challenging to disentangle the role of evolutionary history in these symbioses from contemporaneous ecological mechanisms (e.g., species interactions, environmental conditions, dispersal limitation). Previous studies across a variety of plant‐associated microbiomes have found that host species identity or phylogenetic relatedness often plays a significant role in structuring the host microbiome. For example, host species identity can drive the structure of foliar bacterial communities among forest tree species (Laforest‐Lapointe et al., 2017) and the composition of pathogenic virus communities among grasses (Seabloom et al., 2013). The probability that foliar fungal pathogens will spill over onto introduced, non‐native hosts in grassland communities increases with increasing phylogenetic relatedness and population densities of native plant species in the resident community (Parker et al., 2015). By contrast, root‐associated communities may show a reduced effect of host species identity or phylogenetic distance compared to aboveground communities (David et al., 2016; Glynou et al., 2018; Wagner et al., 2016).

Foliar fungal endophytes (FFE) are a major component of the plant microbiome and have recently emerged as a useful model for testing the outcome of plant‐microbial interactions due to their ease of cultivation and relative tractability under laboratory conditions (Hawkes & Connor, 2017). FFE have existed within plant hosts since land colonization (Krings et al., 2007) and can act as mutualists, commensalists, pathogens, or saprotrophs (Busby et al., 2016). Most FFE colonize via environmentally transmitted propagules from wind, rain, or the previous season's plant litter (Christian et al., 2015) and have both high spatial (David et al., 2016) and temporal (Bowsher et al., 2020) turnover. Previous research has provided contrasting evidence of both FFE host specificity (i.e., FFE community differences among host species or genotypes) and host phylogenetic drivers (i.e., correlation between FFE community distance and host phylogenetic distance). For example, FFE showed host‐specificity to *Populus* genotypes (Bálint et al., 2015) and herbaceous plant species (Gange et al., 2007), while spatial distance was a stronger determinant of FFE community structure than host identity in other reports (i.e., *Picea* sp. and tropical forest grasses; Eusemann et al., 2016; Higgins et al., 2014). Similarly, one recent study found that FFE community structure was significantly related to host phylogeny among *Ficus* tree species in a mixed botanical garden (Liu et al., 2019), but no evidence for host phylogenetic drivers was found among families of trees in a tropical forest (Vincent et al., 2016). Thus, experiments that control for host phylogeny, but minimize broad regional differences in spatial and temporal sampling of host individuals, would be a strong test for the relative importance of host phylogeny versus local ecological conditions on the distribution of FFE.

Here we investigated the relative roles of host phylogenetic relatedness and spatial distance between hosts on FFE community richness, diversity, and structure in a multispecies common garden. The use of a common garden framework allowed us to control for potentially confounding spatial and temporal factors, such as regional‐scale changes in environmental and climatic conditions, long‐distance dispersal limitation of FFE, and host age, by spatially randomizing all species under similar environmental conditions (Kawecki & Ebert, 2004). We predicted that FFE community structure would differ significantly among host species (Laforest‐Lapointe et al., 2017) and that FFE community dissimilarity would be positively associated with phylogenetic divergence among host taxa (Liu et al., 2019). Similarly, we expected to see a positive, though weaker, effect of spatial distance with FFE community dissimilarity across the plots in the common garden due to the limited distance between plots. Lastly, we predicted that FFE community richness and diversity would differ among host species in the common garden reflecting inherent differences among hosts in their suitability as habitat for FFE taxa (Gange et al., 2007).

## 2 MATERIALS AND METHODS

### 2.1 Study system

We analyzed FFE communities of native perennials within the family Asteraceae using a host phylogenetic framework. With approximately 24,000 recognized species, the Asteraceae is the largest family of vascular plants and represents 10% of all flowering plants (Funk et al., 2009). Geographic spread and adaptive radiations out of South America have extended the range of the Asteraceae to every continent except Antarctica (Funk et al., 2009). Previous classifications segregated the Asteraceae into five major lineages, but more recent phylogenetic analyses support twelve subfamilies, with the most basal clades being either wholly endemic, or largely constrained, to South America (Panero & Funk, 2008). FFE have previously been cultured from species of Asteraceae (Christian et al., 2016), and a comparison of FFE communities between two host species from co‐occurring genera of Asteraceae growing in southern England showed host‐specific differences in FFE diversity and abundance (Gange et al., 2007).

### 2.2 Species selection and plant propagation

**FIGURE 1:**
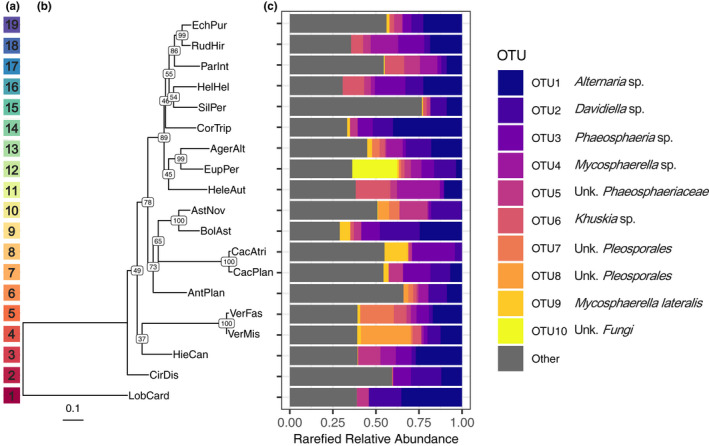
Relative abundance of top 10 most abundant OTUs differs among host species. (A) Color code corresponding to the phylogenetic tree and relative phylogenetic distances among host species—*as a reference for* *Figure* *2*. (B) Phylogenetic tree of the 19 host species using maximum likelihood methods (nodes labeled with bootstrap confidence values). Host species codes are given in Table 1. (C) Rarefied relative abundance for the top ten most abundant fungal OTUs and all other OTUs are shown as proportions, where each colored bar represents a different OTU. Best match names for each of the top ten OTUs are also shown. For “unknown” taxonomic levels, the abbreviation “Unk.” is used

Individual plants from 28 species of Asteraceae spanning three subfamilies, seven tribes, and 22 genera (Funk et al., 2009) were planted into each of six replicated plots within the common garden. Additionally, two outgroup species were included from the Campanulaceae, a sister family to the Asteraceae within the order Asterales (Table 1; Figure 1b). When selecting species, priority was given to species that (a) had more highly resolved taxonomic placement, (b) were perennial and native to Indiana (IN), USA, and (c) had provinciality in southern IN (which is biogeographically divergent from northern IN) or that had a contiguous distribution across the entire state. Seeds of all species were purchased from Prairie Moon Nursery (Winona, MN; seed lot numbers provided in Table A1). Because of the wide variation in seed size, porosity, and other seed surface characteristics among species, it was not logistically tractable to optimize surface sterilization techniques for all 30 species. Additionally, initial tests indicated potential seed death due to bleach for highly porous seeds. Therefore, we did not surface sterilize seeds. Seeds were cold stratified as necessary according to vendor specifications using pasteurized sand and sterile water. The seeds were then germinated under common greenhouse conditions in 20.3 × 20.3 cm square flats filled with commercial potting mix (Metro‐Mix 360, Sun Gro Horticulture), which was first sterilized by autoclaving for 4 hr. Two to three weeks after germination, individual seedlings were transplanted into containers™ (Stuewe & Sons, Yellow (U), RLC3) filled with sterilized Metro‐Mix. After 5–6 weeks of growth under uniform greenhouse conditions, plants were then transplanted into the common garden.

**TABLE 1:** Common Garden plant species and taxonomic information

Genus	Species	Host Species Code	Sub‐Family	Tribe	Sub‐Tribe
Plant species sampled for Illumina sequencing					
*Ageratina*	*altissima*	AgerAlt	Asteroideae	Heliantheae Alliance	Eupatorieae
*Antennaria*	*plantaginifolia*	AntPlan	Asteroideae	Gnaphalieae	
*Aster*	*novae‐angliae*	AstNov	Asteroideae	Astereae	
*Boltonia*	*asteroides*	BolAst	Asteroideae	Astereae	
*Arnoglossum*	*atriplicifolium*	CacAtri	Asteroideae	Senecioneae	
*Arnoglossum*	*plantagineum*	CacPlan	Asteroideae	Senecioneae	
*Cirsium*	*discolor*	CirDis	Carduoideae	Cardueae	Carduinae
*Coreopsis*	*tripteris*	CorTrip	Asteroideae	Heliantheae Alliance	Coreopsideae
*Echinacea*	*purpurea*	EchPur	Asteroideae	Heliantheae Alliance	Heliantheae
*Eupatorium*	*perfoliatum*	EupPer	Asteroideae	Heliantheae Alliance	Eupatorieae
*Helenium*	*autumnale*	HeleAut	Asteroideae	Heliantheae Alliance	Helenieae
*Heliopsis*	*helianthoides*	HelHel	Asteroideae	Heliantheae Alliance	Heliantheae
*Hieracium*	*canadense*	HieCan	Cichorioideae	Cichorieae	Hieraciinae
*Lobelia*	*cardinalis*	LobCard	(*outgroup*) Campanulaceae Family		
*Parthenium*	*integrifolium*	ParInt	Asteroideae	Heliantheae Alliance	Heliantheae
*Rudbeckia*	*hirta*	RudHir	Asteroideae	Heliantheae Alliance	Heliantheae
*Silphium*	*perfoliatum*	SilPer	Asteroideae	Heliantheae Alliance	Heliantheae
*Vernonia*	*fasciculata*	VerFas	Cichorioideae	Vernonieae	Vernoniinae
*Vernonia*	*missurica*	VerMis	Cichorioideae	Vernonieae	Vernoniinae
Plant species not sampled for Illumina sequencing					
*Eupatorium*	*coelestinum*	EupCoel	Asteroideae	Heliantheae Alliance	Eupatorieae
*Helianthus*	*grosseserratus*	HeliGro	Asteroideae	Heliantheae Alliance	Heliantheae
*Liatris*	*spicata*	LiaSpic	Asteroideae	Heliantheae Alliance	Latrinae
*Lobelia*	*spicata*	LobSpic	(*outgroup*) Campanulaceae Family		
*Prenanthes*	*alba*	PreAlba	Cichorioideae	Cichorieae	Hypochaeridinae
*Prenanthes*	*racemosa*	PreRace	Cichorioideae	Cichorieae	Hypochaeridinae
*Ratibida*	*pinnata*	RatPin	Asteroideae	Heliantheae Alliance	Heliantheae
*Solidago*	*nemoralis*	SolNem	Asteroideae	Astereae	Solidagininae
*Verbesina*	*alternifolia*	ActAlt	Asteroideae	Heliantheae Alliance	Heliantheae
*Verbesina*	*helianthoides*	VerbHel	Asteroideae	Heliantheae Alliance	Heliantheae
*Vernonia*	*altissima*	VerAlt	Cichorioideae	Vernonieae	Vernoniinae

Note

Taxonomic information is provided where applicable. For some species, plant sub‐tribe is not defined.

### 2.3 Common garden

**FIGURE A1:**
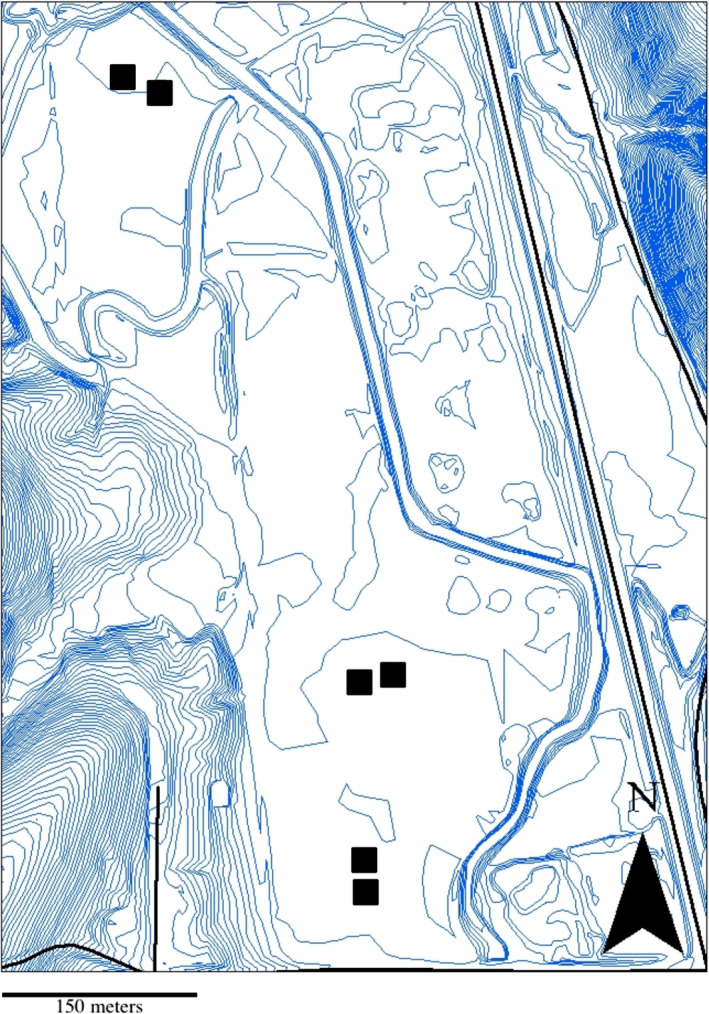
Topographic map showing the layout of the common garden plots at Bayles Rd., Bloomington, IN. Black squares show the location of the six replicated plots. Blue contour lines indicate 0.61m elevational changes

The common garden was established in April and May 2014 at the Indiana University Research and Teaching Preserve Bayles Road field site in Bloomington, IN (N 39.217, W −86.540; Figure A1). Bayles Road is a former agricultural field that underwent active tilling and crop rotation from the 1950–1990s but has since been used for ecological research. The local plant community at Bayles Road includes grasses (e.g., *Andropogon virginicus*, *Lolium arundinaceum*, *Poa pratensis*, *Sorghum halapens*, *Tridens flavus*), forbs (e.g., *Asclepias syriaca*, *Rubus* spp., *Toxicodendron radicans*), and several species of trees, which border the property (e.g., *Acer negundo*, *Acer saccharinum*, *Liriodendron tulipifera*, *Platanus occidentalis*). The surrounding vegetation at the site included many genera of native Asteraceae (e.g., *Aster*, *Ambrosia*, *Cirsium*, *Erigeron*, *Packera*, *Solidago*, *Verbesina*, *Vernonia*), that could serve as potential FFE inoculum sources for experimental plants. To reduce the growth of weeds and to facilitate seedling establishment, all common garden plots were first mowed, sprayed twice over 2 days with a glyphosate‐based herbicide at a 1.5% rate (Aquamaster; Monsanto Co.), and then tilled two times after plant dieback. To further minimize weed growth, black landscaping cloth (Hummert International) was spread across the tilled soil and secured using 15.2 cm metal staples in all plots. A total of six 9.1 × 7.0 m plots were established in a paired plot design, with the three sets of paired plots located at the northern, central, or southern regions of the Bayles Road field site (Figure A1). Paired plots provided replication to separate spatial effects from local site effects. Each plot within a pair was spaced at least 13 m apart, and the two most distant plots were spaced 648 m apart. Planting arrangement for replicate individuals of all 30 species was randomized across plots, with three replicate individuals per species per plot in a full‐factorial experimental design (i.e., 3 replicates × 30 host species × 6 plots; Total *N* = 540). All plants were transplanted into the common garden plots 3 weeks postherbicide spray in a rectangular grid, with 0.91 m spacing lengthwise and 0.76 m spacing crosswise between plants. Immediately after transplantation, each plant received 0.5 L of liquid fertilizer (3.91 ml per L‐H_2_O, Jack's Classic All Purpose 20–20–20, JR Peters Inc., Allentown PA) to improve initial establishment in the field, after which no additional watering or fertilization was applied. As necessary, plots were hand‐weeded and individual transplants were protected from small mammal herbivory with wire mesh enclosures (a cylinder 15 cm tall, with 1 cm^2^ mesh size) until established.

### 2.4 Leaf collection

**FIGURE A2:**
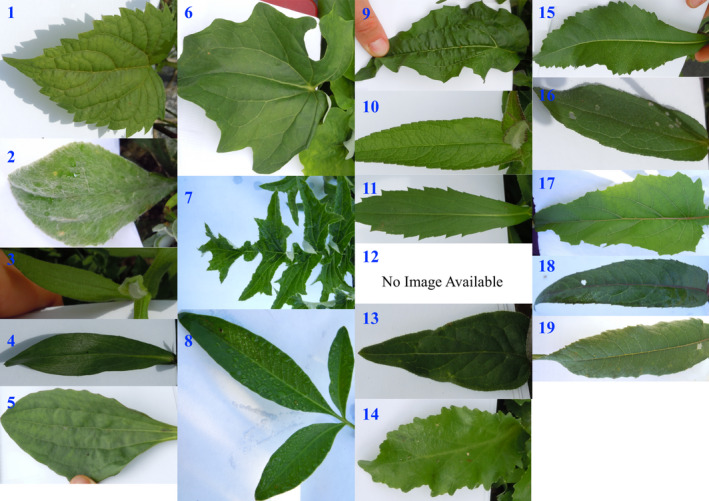
Depiction of variation in leaf morphology across Asteraceae species sampled from the common garden. Image scale and light intensity across panels is not comparable. From 1–19: (1) *Ageratina altissima*, (2) *Antennaria plantaginifolia*, (3) *Aster novae‐angliae*, (4) *Boltonia asteroides*, (5) *Arnoglossum plantagineum*, (6) *Arnoglossum atriplicifolium*, (7) *Cirsium discolor*, (8) *Coreopsis tripteris*, (9) *Echinacea purpurea*, (10) *Eupatorium perfoliatum*, (11) *Helenium autumnale*, (12) *Heliopsis helianthoides*, (13) *Hieracium canadense*, (14) *Lobelia cardinalis*, (15) *Parthenium integrifolium*, (16) *Rudbeckia hirta*, (17) *Silphium perfoliatum*, (18) *Vernonia fasciculata*, (19) *Vernonia missurica*

After 3 months of growth and exposure to natural sources of endophyte inocula, leaves were harvested for FFE sampling in September 2014. We used culture‐independent Illumina sequencing to identify FFE taxa across hosts in our common garden, independent of visible symptoms of colonization. A subset of 19 species were chosen (18 Asteraceae, 1 Campanulaceae; Table 1) due to logistical and financial constraints, with one replicate individual per species per plot randomly selected (*N* = 6 per species and *N* = 114 total plants sampled). To minimize differences in sampling method among the 19 host species, which varied widely in height, growth architecture, and leaf size (see Figure A2), three leaves per plant (or leaf sub‐sections from large‐leaved plant species) were selected from mid‐stem height. Leaf samples were then stored at 4°C until processing, which occurred within 24 hr of collection. To minimize differences in leaf sterilization efficiency across species, we standardized the size and shape of leaf fragments sterilized (nine haphazardly selected 0.5 × 0.5 cm square fragments). Leaf fragments were surface sterilized for 3 min in 70% ethanol, 2 min in 0.5% sodium hypochlorite, 1 min in sterile water, and then allowed to air dry for 1 min (Mejía et al., 2008). Surface‐sterilized leaf fragments were then placed in sterile 2‐ml microcentrifuge tubes, flash‐frozen in liquid nitrogen, and stored at −80°C. Two additional leaf disks per plant (1 cm diameter) were sampled from similarly aged, proximate leaves 3–4 weeks after collecting leaf samples for Illumina sequencing to determine leaf mass per area (LMA). Leaf disks were oven‐dried at 60°C for 3 days and weighed.

### 2.5 Molecular analyses

All nine leaf fragments per individual host were bulked, and DNA was extracted using the PowerPlant^®^ Pro DNA Isolation Kit (MO BIO Laboratories) following the manufacturer's instructions, save for changes to the tissue homogenization step (MP Biomedicals FastPrep^®^‐24 Tissue Homogenizer; twice at 4 m/s for 60 s). We modified the extraction protocol following manufacturer's instructions to include 40 μl of Phenolic Separating Solution and 250 μl of solution PD3 (see FigShare repository for specific sample IDs) as needed due to low‐quality DNA extractions for certain samples.

Nested PCR was used to improve fungal amplicon yields based on preliminary tests on Asteraceae samples, where a single round of PCR amplification typically failed to yield enough abundant, high quality Illumina reads (Binet et al., 2017). First, primers NSA3 and NLC2 (Martin & Rygiewicz, 2005) were used to amplify an ~1,000 bp region surrounding the internal transcribed spacer (ITS) region (SSU, ITS1, 5.8S, ITS2, and LSU) of the fungal nuclear ribosomal DNA gene via PCR using GoTaq^®^ DNA Polymerase (Promega Corporation) as per the manufacturer's recommendations in a 25 μl reaction with 1 μl of template diluted 1:10 in molecular‐grade water. A Tetrad PTC‐225 Peltier Thermal Cycler (MJ Research) was used for PCR reactions, with the thermal cycler program recommended by Promega: 2.5 min at 95°C, followed by 25 cycles (30 s at 95°C, 30 s at 60.2°C, 45 s at 72°C), then 5 min at 72°C. Amplification of samples and clean negative controls were confirmed using gel electrophoresis. Amplicons were purified using the MicroElute^®^ Cycle‐Pure Kit (Omega Bio‐Tek, Inc.) and sent to the Biosciences Division (BIO) Environmental Sample Preparation and Sequencing Facility (ESPSF) at Argonne National Laboratory (ANL) for sequencing on the Illumina MiSeq platform. At ANL, products of the first PCR were amplified using a modified version of the fungal‐specific ITS1F and ITS2 primer set (Smith & Peay, 2014). The reverse amplification primer also contained a twelve‐bp Golay barcode sequence, which was read using a third sequencing primer in an additional cycle (Caporaso et al., 2010, 2012). Each 25 µl PCR reaction consisted of 9.5 µl of molecular‐grade water, 12.5 µl of QuantaBio AccuStart II PCR ToughMix (2× concentration, 1× final), 1 µl Golay barcode tagged Forward Primer (5 µM concentration, 200 pM final), 1 µl Reverse Primer (5 µM concentration, 200 pM final), and 1 µl of template DNA. Amplification was performed as follows: 3 min at 94°C, followed by 35 cycles (45 s at 94°C for, 60 s at 50°C, 90 s at 72°C), 10 min at 72°C.

Amplicon concentrations were quantified using PicoGreen (Invitrogen), pooled at equal‐molar concentrations, cleaned using AMPure XP Beads (Beckman Coulter), and quantified using Qubit (Invitrogen). After quantification, the pool was first diluted to 2 nM, denatured, and then diluted to 6.75 pM with a 10% PhiX spike for paired 251‐nuceotide read sequencing on the Illumina MiSeq platform. To exclude the PhiX control reads from downstream analysis, the first read of all read pairs was mapped against a PhiX reference using BWA (v.0.6.2‐r126; Li & Durbin, 2009). All reads that successfully mapped to the PhiX reference were discarded. A custom Perl script was used to demultiplex samples from the pooled sequence data. All resulting paired forward and reverse sequence reads were merged to create a single contig using Mothur (v.1.37.1; Schloss et al., 2009) for workflow management (9,001,866 total reads pre‐filtering). Resulting contigs with ambiguous bases, or with lengths greater than 350 bp, were removed as part of quality filtering. Chimeras were removed using UCHIME (v.4.2.40; Edgar et al., 2011).

Two samples failed to amplify, while six samples had read counts less than 10,000 and were thus removed prior to clustering. For the community analyses, this left *N* = 106 individual hosts across 19 plant species. Reads were clustered by sequence similarity into Operational Taxonomic Units (OTUs) using AbundantOTU+ (v.0.93b; Ye, 2010) at 95% (5,983,719 remaining reads; 559 OTUs) and 97% (5,417,921 remaining reads; 669 OTUs). Statistical analyses did not differ substantially between the 95% and 97% sequence identity datasets; therefore, only results for the 95% OTU threshold are presented here. Putative names were assigned to each OTU using the RDP naïve Bayesian classifier (v.2.12) and the Warcup fungal ITS database (Deshpande et al., 2015). Results from this classification, along with confidence thresholds for each level in the taxonomic hierarchy, are available through the FigShare repository.

### 2.6 Phylogenetic inference

To generate a measure of phylogenetic relatedness in our analysis of FFE communities, a single locus phylogeny was reconstructed for all 19 plant species using plant nuclear ITS sequences retrieved from GenBank (see Table A2 for accession numbers & species names). ITS sequences show moderately high levels of sequence divergence and have proven useful for phylogenetic analysis at lower taxonomic levels (i.e., sub‐families, tribes, genera; Feliner & Rosselló, 2007; Li et al., 2015), including in the Asteraceae (Gemeinholzer et al., 2006). Species lacking available sequences in GenBank were replaced with available ITS sequences from congeneric species (*n* = 3), or contribal species (*n* = 3), based on previous phylogenetic inference in the Asteraceae (Fu et al., 2016; Panero & Funk, 2008; Schmidt & Schilling, 2000; Urbatsch et al., 2000). Plant sequences were aligned using Muscle version 3.8.31 (Edgar, 2004) and converted to PHYLIP format. Maximum likelihood (ML) analysis was conducted using RAxML HPC‐PThreads v.8.2.12 (Stamatakis, 2014) and a single step (‐f a code, 10,000 bootstrap replicates) by applying ML tree search and rapid bootstrapping. *Lobelia cardinalis* (Campanulaceae) was set as the outgroup species (‐o code). We chose the best supported bipartitioned tree calculated from the generalized time reversible (GTR) substitution model with a GAMMA model of rate heterogeneity. The topology of the best tree (Figure 1a, b) agreed with previously published phylogenies in the Asteraceae (Panero & Funk, 2008). The final best scoring ML tree was used to provide a measure of phylogenetic distance between all species pairs in our data set.

### 2.7 Statistical analyses

All statistical analyses were run in R (v.3.5.2; R Core Team, 2020). Before conducting all analyses, we subsampled (rarefied) to 15,514 reads per sample, which was the sequencing depth of the lowest sample after quality filtering (Bowsher et al., 2020). The best scoring ML tree was imported into R using the “ggtree” package (Figure 1a,b; Yu et al., 2017).

#### 2.7.1 FFE community structure analyses

We used the Bray–Curtis dissimilarity index to test for differences in FFE community structure using a permutational multivariate analysis of variance (PERMANOVA) and a marginal sum of squares method to compute pseudo *F*‐statistics for hypothesis testing (“adonis2” function, “vegan” package, 3,000 permutations; Oksanen et al., 2017). For the FFE community structure analysis, two predictor variables were tested as categorical variables (host species identity and common garden plot), while LMA was tested as a covariate and continuous variable. The interaction between host species identity and LMA was insignificant and thus not included in the final model. Each FFE's abundance was relativized by the rarefied number of reads per host (Legendre & Gallagher, 2001). As an additional test for the effect of host phylogeny on FFE communities, separate PERMANOVAs were performed for host tribe (seven tribes plus Campanulaceae outgroup) and host sub‐family (three sub‐families plus Campanulaceae outgroup), in place of host species.

Differences in FFE community structure were visualized using principal coordinates analysis (PCoA). To visualize the roles of host species identity and host phylogenetic relationships in structuring FFE communities, color was assigned to each sample point in the PCoA plots based on the phylogenetic tree (Figure 1a,b). To simplify the presentation of host specificity in structuring FFE communities, only the ellipses identifying the centroid and standard error of the seven Asteraceae tribes and the Campanulaceae outgroup species are presented, in lieu of 19 host species ellipses. To determine how much variance was explained by each of the predictor variables (host species identity, common garden plot, and LMA), we performed a variance partitioning analysis (Legendre & Gallagher, 2001) with the Bray–Curtis community distance matrix as the response variable (“varpart” function, “vegan” package). Constrained dbRDA followed by a pseudo‐*F* test was used to assess significance of each predictor variable (“capscale function," “vegan” package; Oksanen et al., 2017). Due to missing LMA data for nine host plants, only *N* = 97 plants were included in the community richness, diversity, and structure analyses.

#### 2.7.2 FFE richness & diversity analyses

**FIGURE A3:**
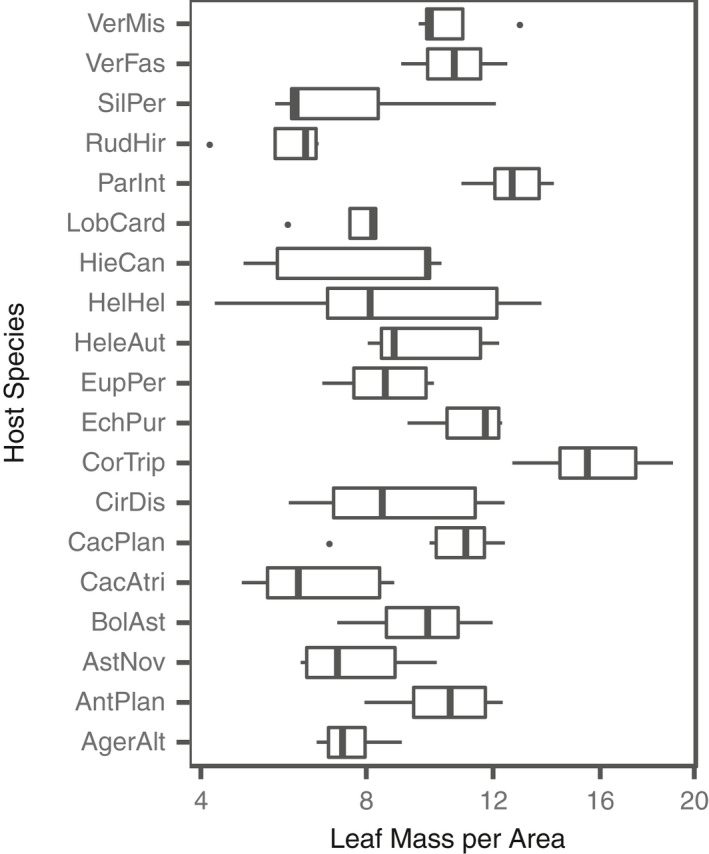
Average Leaf Mass per Area (LMA) varied among host species. Boxplots are displayed for each species. The *x*‐axis is shown on a square‐root scale. Species are sorted alphabetically from bottom to top. The host species codes appear as in Table 1

**FIGURE A4:**
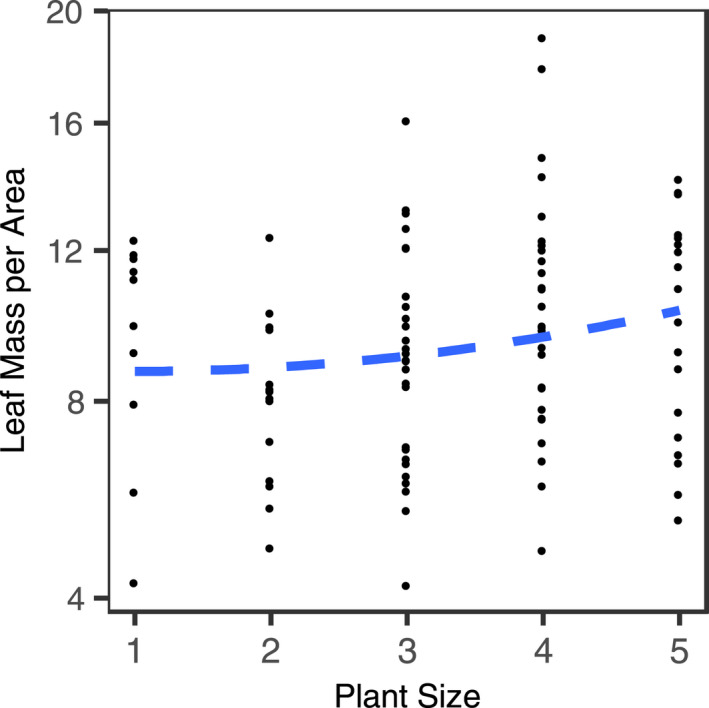
Quadratic regression (blue dashed line) of Leaf‐Mass Area (LMA) against individual host size. Each point represents a single host individual. Individual plant size was qualitatively assigned to one of five size classes at the time of leaf collection. The *y*‐axis is shown on a square‐root scale. (Quadratic Term *p* = 0.0211; Linear Term *p* = 0.0033)

We used linear models to test how FFE OTU richness and diversity varied among individual plants, the unit of replication in this study. For these models, we used LMA to standardize FFE richness and diversity per unit leaf mass, because while the same area of leaf tissue was sampled for Illumina sequencing, leaf mass varied among host individuals and species (Figure A3). LMA was also a significant correlate of individual plant size (*Adj. R* ^2^ = 0.64 from model including plant size, host species and plots; Figure A4). Linear models tested whether richness and diversity varied among host species and plots in the common garden. FFE richness was square‐root transformed to meet assumptions of normality.

#### 2.7.3 FFE community distance analyses

**FIGURE A5:**
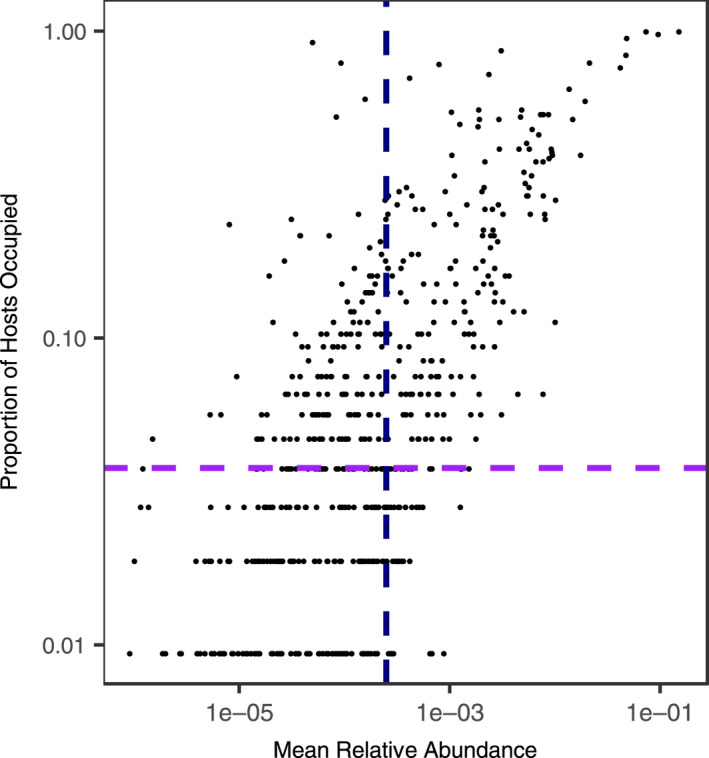
Occupancy‐Abundance Plot. The proportion of host individuals each FFE occurred in plotted against the average relative abundance of each FFE across all sampled hosts (both log_10_‐transformed). To the right of the vertical‐dashed line (black) indicates FFE that had an average relative abundance of greater than 0.025%, while above the horizontal dashed line (purple) indicates FFE that occupied greater than 4 out of 106 host individuals

A multivariate statistical framework was used to test the association of FFE community structure with host species phylogeny and plot location within the common garden (*N* = 106 plants). Specifically, we performed partial Mantel tests to examine the correlation between the rarefied and normalized Bray–Curtis dissimilarity matrix with pairwise host species phylogenetic distance while controlling for pairwise spatial distance among plots, and vice versa. A null distribution drawn from 9,999 permutations of the Bray–Curtis matrix was used to test for statistical significance. Pairwise phylogenetic distance among all species pairs was estimated using the “cophenetic” function of tree branch length in R. The distance between plots was always substantially greater than the distance between plants within plots. Therefore, pairwise spatial distance between the centroid of all field plots in the common garden was calculated using Google Earth v.7.1.7.2602. Additionally, we tested whether core or rare taxa were drivers of distance‐decay patterns. We created occupancy–abundance plots (Figure A5; Shade et al., 2018) using prevalence and mean relative abundance across the dataset. Core taxa were selected on the criteria that they were (a) present in more than four host plants and (b) had a mean relative abundance greater than 0.025%. This partitioned the full FFE community matrix into 178 core and 380 rare fungal OTUs.

## 3 RESULTS

### 3.1 Basic sequencing results

The quality‐filtered sequence dataset, based on 106 plant samples, contained 558 fungal OTUs (after rarefaction) generated from 5,983,719 ITS1 reads. The average sequencing depth per sample before rarefaction was 56,450 reads and ranged from 15,514 to 88,922. The majority of identified fungal OTUs belonged to the phylum Ascomycota (79.1%), while 12.3% belonged to the Basidiomycota. Additionally, fungal OTUs represented 29 orders where the five most abundant orders were Pleosporales (166 OTUs), Capnodiales (78 OTUs), Trichosphaeriales (41 OTUs), Xylariales (34 OTUs), and Tremellales (27 OTUs). 110 OTUs, comprising 10.3% of total sequencing reads, could not be identified to the order level. The ten most common OTUs represented 56.1% of the total sequence reads and varied in normalized relative abundance among the 19 host species (Figure 1c).

### 3.2 FFE community structure, richness, and diversity

**FIGURE 2:**
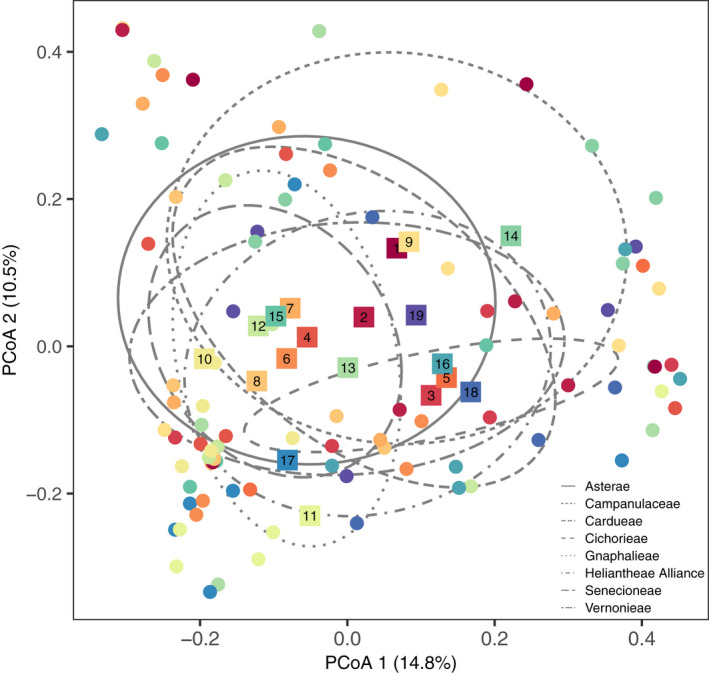
FFE community structure varied by host species and tribe. The location of each host individual in ordination space is denoted by a filled circle, while the centroid, or average PCoA coordinates, of all 19 host species are shown as filled, numbered squares. Host species are color coded according to their phylogenetic relationship, where cooler colors represent more derived lineages and warmer colors represent more basal lineages*—as shown in* *Figure* *1a*. The ellipses represent the centroid and standard deviation for each of the seven host tribes and the Campanulaceae family (i.e., *Lobelia cardinalis*) outgroup

**FIGURE A6:**
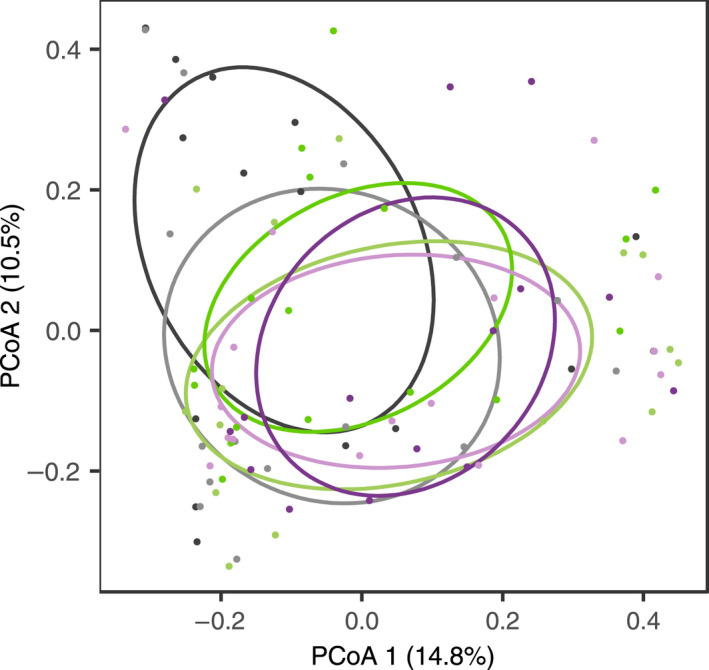
FFE community structure did not significantly vary among plots in the common garden. Ellipses depict the centroid and standard deviation for common garden plots. The two grey‐shaded ellipses represent plots 1 & 2, the two green‐shaded ellipses represent plots 3 & 4, and the two purple‐shaded ellipses represent plots 5 & 6. The location of each host individual in ordination space is denoted by a filled circle and is color‐coded in kind with the ellipses

After 3 months of exposure to natural inoculum sources, there was a significant effect of host species identity on the structure of FFE communities (pseudo‐*F*_18,72_ = 1.26, *p* = 0.0033; Figure 2; see Figure 1a,b for phylogenetically based color reference). There was also a significant effect of host tribe on the structure of FFE communities (pseudo‐*F*_7,83_ = 1.30, *p* = 0.0143; Figure 2), but there was no significant effect of host subfamily (*p* = 0.5448). There was a non‐significant trend toward differences in FFE community structure between different common garden plots (pseudo‐*F*_5,72_ = 1.23, *p* = 0.06390; Figure A6). There were no significant differences among host plants with differing LMA (*p* = 0.2422). A variance partitioning analysis demonstrated that host species identity explained a significant amount of variation in FFE community structure (4.85%; *p* = 0.002). Neither common garden plot identity (1.40%; *p* = 0.078) or LMA significantly explained any variation in FFE community structure (0.23%; *p* = 0.256).

**FIGURE A7:**
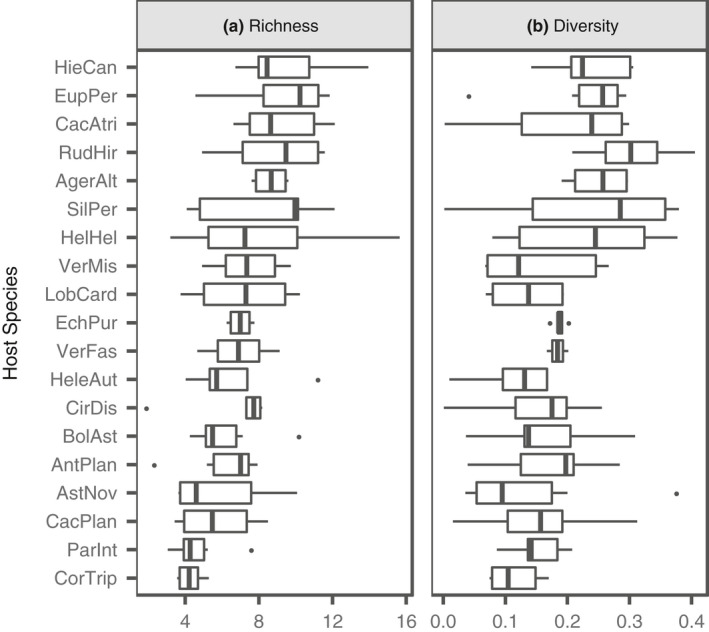
FFE community (a) richness and (b) diversity did not vary significantly by host species. Boxplots are displayed for each species. For both panels, species have been sorted from least to highest FFE diversity. The host species codes appear as in Table 1. Richness and diversity have both been standardized by Leaf Mass per Area

**FIGURE A8:**
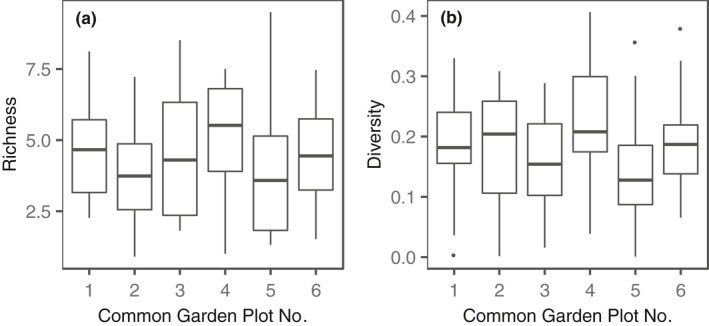
FFE community (a) richness and (b) diversity did not vary significantly among common garden plots. Boxplots are displayed for each common garden plot. Richness and diversity have both been standardized by Leaf Mass per Area

The average OTU richness per host was 40 OTUs after rarefying and ranged from 6 to 94 OTUs per host. However, FFE OTU richness did not differ among host species (*p* = 0.1830; Figure A7a) or among plots in the common garden (*p* = 0.2711; Figure A8a). Similarly, though the average OTU diversity per host was 1.65, it also did not differ among host species (*p* = 0.1439; Figure A7b) or among common garden plots (*p* = 0.2759; Figure A8b).

### 3.3 FFE community distance

**FIGURE 3:**
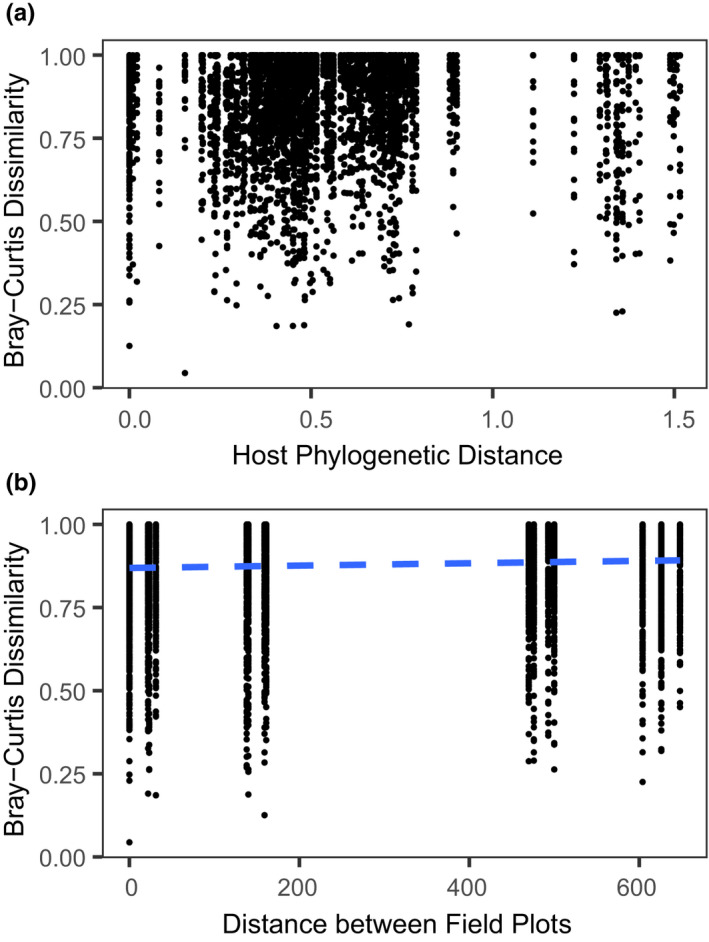
FFE community dissimilarity A) did not significantly correlate with host phylogenetic distance but B) did significantly correlate with spatial distance between common garden field plots. The regression line (blue‐dashed lines) characterizes the relationship between pairwise FFE community distance with pairwise distance between plots in the common garden. Each point represents a single pairwise comparison

Phylogenetic distance among hosts ranged from 0 for individuals of the same species to 1.52 branch length units for individuals of the two most divergent species (i.e., *L. cardinalis* and *Cacalia plantaginea*; Figure 1b). The pairwise spatial distance between plots in the common garden ranged from 0 to 648 m. Results of the partial Mantel test showed that dissimilarity of FFE communities among hosts was not significantly correlated with host phylogenetic distance (*p* = 0.1719; Figure 3a). By contrast, pairwise distance between plots within the common garden was significantly and positively correlated with the dissimilarity of FFE communities among hosts, although it explained relatively little of the variation (*r* = 0.0610; *p* = 0.0299; Figure 3b). When the FFE community was divided into core and rare components, the core community displayed significant spatial distance decay within the common garden (*r* = 0.0683; *p* = 0.0183), while the rare community did not (*p* = 0.0937). Neither the core nor rare community was significantly correlated with host phylogenetic distance (core *p* = 0.1509; rare *p* = 0.5119).

## 4 DISCUSSION

Our study represents the first to investigate host phylogenetic relationships for FFE microbiota using a common garden approach, where individual plants from different species of the same age were spatially randomized across replicate plots within the same local habitat. We found that FFE communities differed significantly among host species and host tribes but that, contrary to our original prediction, pairwise phylogenetic distance between host species was not significantly correlated with FFE community dissimilarity between individual hosts. By contrast, FFE community dissimilarity did show modest increases with increasing spatial distance in the common garden over the limited spatial scale of 648 m. Overall, our results do not support the hypothesis that more closely related Asteraceae species within a common garden share more similar FFE microbiomes, but do suggest that other differences among host species, unaccounted for by host phylogenetic distance, lead to divergence in FFE communities among host species.

Our common garden experiment was established with the explicit purpose of maximizing local effects (e.g., small‐scale environmental conditions and local inocula sources), while minimizing regional effects (e.g., geographic differences in surrounding vegetation, climatic conditions, soil types) on FFE community structure. As a result, host species in the common garden shared many of the same FFE OTUs (see overlap in Figure 2), despite exhibiting significant species‐specific differences. Given the large differences across plant species in leaf traits (Figure A2), plant architecture, chemistry, phenology, and other phenotypic traits, it is perhaps surprising that we did not see more variation in FFE community structure among species. Future research should examine how host traits interact with environmental factors to influence microbial colonization and FFE community assembly. For example, significant variation in leaf secondary chemistry is a well‐documented feature of the Asteraceae family that is not well resolved by phylogenetic relationships (Calabria et al., 2007) and that could influence colonization and persistence of specific microbial taxa (Christian et al., 2020). Micro‐environmental differences among leaves sampled from different host individuals (e.g., leaf age, variable UV exposure; Osono & Mori, 2003) could be another source of unexplained variation in FFE community structure identified in our study. In addition, recent evidence indicates that microbial colonization from conspecific and heterospecific neighboring plants can also affect microbial community structure in mixed forests (Laforest‐Lapointe et al., 2017). We did not quantify variation in the vegetation surrounding the common garden plots or within the common garden itself, but the neighboring vegetation may have influenced microbial community structure via short‐distance dispersal or via priority effects of initial colonizers onto newly emerged leaves (Adame‐Álvarez et al., 2014).

Contrary to our original prediction, we did not find a significant role for phylogenetic relatedness in determining the structure of FFE communities. Moreover, relatively little of the observed variation in FFE community structure was explained by any of the factors tested here. Host phylogenetic relatedness has often been used as a proxy for understanding complex ecological and evolutionary processes because it is simpler to measure than an array of functional traits and relatively inexpensive with the increasing availability of genetic sequence data (Cavender‐Bares et al., 2009). The genotypic and phenotypic divergence among host species underlying phylogenetic relationships is complex and undoubtedly includes a wide array of traits that differentially influence colonization success of specific microbial taxa (Liu et al., 2019). For example, research on plant–pathogen interactions suggests that more closely related hosts share similar genetic pathways for cellular recognition of proteins and effector molecules during pathogen colonization and resistance (Barrett & Heil, 2012). Our results stand in contrast to previous studies demonstrating significant signatures of host phylogenetic relationships for foliar fungal pathogens (Gilbert & Webb, 2007; Parker et al., 2015), root endophytic fungi (Wehner et al., 2014), arbuscular mycorrhizal fungi (Anacker et al., 2014), and FFE communities among *Ficus* species in a botanical garden (Liu et al., 2019). The Asteraceae represent a speciose plant family that has evolved more recently than the *Ficus* clade (Stevens, 2019). Thus, there may be less evolutionary divergence in traits related to asymptomatic FFE colonization for the Asteraceae relative to *Ficus*. On the other hand, our insignificant phylogenetic results were consistent with studies on belowground fungi and bacteria (David et al., 2016; Wagner et al., 2016) and FFE from host systems of differing phylogenetic breadth (Vincent et al., 2016).

Disentangling local, host‐based processes, such as genotypic and phenotypic differences among individual plants, from spatial processes, such as dispersal, remain a challenge in studies of microbiome assembly and function. For example, one study at the landscape level showed that FFE community structure varied predictably across a 400‐km precipitation gradient but did not significantly differ between two congeneric grass species (Giauque & Hawkes, 2013). While in another study, where both a very broad phylogenetic and spatial breadth of host plants was compared (i.e., angiosperms to bryophytes, across continents), host identity and host genetic distance were the dominant drivers of FFE community structure and dissimilarity while spatial drivers were not detected (U'Ren et al., 2019). Here we detected a significant spatial effect on FFE community structure across 648 m, even while local climatic and soil factors were relatively constant (i.e., less than 1m elevational change across plots, homogeneous soil from a former agricultural field with decades of tilling). Micro‐environmental differences across plots were not measured here (e.g., soil moisture, surrounding vegetation, wind speed), but could be incorporated into future studies to better tease apart small‐scale drivers of FFE community assembly. Similarly, to separate local and regional influences on FFE communities from host genetic effects, experimental plantings of species or genotypes across multiple spatial and environmental scales, followed by microbiome characterization, could be implemented. For example, several genotypes within a group of host species that vary in specific leaf traits, or immune responses, could be transplanted across soil fertility or spatial distance gradients.

Our results likely represent a conservative assessment of the relative importance of host identity and spatial distance in driving FFE communities. Specifically, the restriction of hosts to a single plant family, combined with the relatively rapid speciation of the Asteraceae overall, could have led to an underestimation of host identity importance relative to spatial scale. Additionally, inclusion of negative and positive controls in our sequencing efforts (Nguyen et al., 2015) may have increased the confidence in community differences between host species by more accurately determining the presence or absence of OTUs between samples (Palmer et al., 2018). It is also possible that our PCR conditions (e.g., nested PCR) led to a reduced pool of fungal species for comparison across hosts (Yu et al., 2015). However, it is important to note that studies comparing environmental or spatial factors with host genetic identity in FFE often find a relatively low contribution of host genetic identity (Bálint et al., 2015; Lamit et al., 2014; Whitaker et al., 2018).

In conclusion, our results provide insights and raise additional questions about the assembly and structure of host‐associated microbiota across phylogenetically divergent host lineages (Clay & Schardl, 2002; Ley et al., 2008; Russell et al., 2017). Most previous research on the role of host phylogenetic relatedness in structuring microbial communities has relied on field sampling of natural populations and communities where host taxa occurred in different locations or microenvironments (Eusemann et al., 2016; Tedersoo et al., 2013; Vincent et al., 2016; Wehner et al., 2014) and thus did not control for regional and temporal drivers of microbial community assembly. Our study controlled for those factors, and we demonstrate that host species identity and spatial distance are significant, albeit modest, drivers of FFE community structure.

## CONFLICT OF INTEREST

We have no competing interests to declare.

## AUTHOR CONTRIBUTIONS

**Briana K. Whitaker:** Conceptualization (equal); data curation (lead); formal analysis (lead); funding acquisition (equal); investigation (equal); methodology (equal); project administration (lead); writing – original draft (lead); writing – review and editing (lead). **Natalie Christian:** Conceptualization (equal); formal analysis (supporting); investigation (equal); methodology (equal); writing – review and editing (equal). **Qing Chai:** Conceptualization (supporting); investigation (equal); methodology (equal); writing – review and editing (supporting). **Keith Clay:** Conceptualization

(equal); funding acquisition (equal); investigation (equal); project administration (equal); writing – review and editing (equal).

## Data Availability

Raw data and R statistical code are available from Figshare (https://doi.org/10.6084/m9.figshare.11407332). Consensus gene sequences are available through NCBI MW157379 ‐ MW157937.

## 1

**TABLE A1:** Seed lot codes and geographic origin within the US for all plant species used in the common garden

Item	Description *[alternate names in brackets]*	Lot	Origin
ACT02F	*Actinomeris [Verbesina] alternifolia*	AE2476R	Midwest
ANT04F	*Antennaria plantaginifolia*	PX1099R	Winona Co. MN
AST18F	*Aster [Symphyotrichum] novae‐angliae*	WW3089R	Allamakee Co. IA
BOL02F	*Boltonia asteroides*	PX2592R	Central IL
CAC02F	*Cacalia [Arnoglossum] atriplicifolia*	CN3158R	Jackson Co. IL
CAC10F	*Cacalia [Arnoglossum] plantaginea*	PX10878Q	Cook Co. IL
CIR02F	*Cirsium discolor*	UF1121R	S. Central WI
COR06F	*Coreopsis tripteris*	CN3162R	Jackson Co. IL
ECH08F	*Echinacea purpurea*	PM2855R	Unknown
EUP03F	*Eupatorium coelestinum*	HF11572Q	Logan Co. IL
EUP06F	*Eupatorium perfoliatum*	PX11491Q	Sherb Co. MN
EUP10F	*Eupatorium rugosum [Ageratina altissima]*	PMP0307	Unknown
HEL02F	*Helenium autumnale*	PX2423R	Winona Co. MN
HEL44F	*Helianthus grosseserratus*	PM1054H	Whiteside Co. IL
HEL82F	*Heliopsis helianthoides*	PM52967R	Unknown
HIE02F	*Hieracium canadense*	PMP0342	Unknown
LIA14F	*Liatris spicata*	HF2982R	Will Co. IL
LOB02F	*Lobelia cardinalis*	ND3071R	Buffalo Co. WI
LOB10F	*Lobelia spicata*	AE2537R	Winona Co. MN
PAR02F	*Parthenium integrifolium*	HF10823Q	LaSalle Co. IL
PRE02F	*Prenanthes alba*	MX10114Q	Winona Co. MN
PRE10F	*Prenanthes racemosa*	TW922J	Grant Co. SD
RAT04F	*Ratibida pinnata*	WW3044R	Winnebago Co. IA
RUD02F	*Rudbeckia hirta*	PM1306R	Madison Co. IA
SIL56F	*Silphium perfoliatum*	PM1287R	Green Co. WI
SOL08F	*Solidago nemoralis*	HF2984R	Logan Co. IL
VER20F	*Verbesina helianthoides*	PMP0337	Unknown
VER10F	*Vernonia altissima*	WL1087A	Delaware Co. IN
VER52F	*Vernonia fasciculata*	PM58K	S.E. MN
VER54F	*Vernonia missurica*	HF11553Q	Menard/ Logan Co. IL

Note

All seeds were purchased from Prairie Moon Nursery.

**TABLE A2:** Plant ITS GenBank accession numbers used in phylogenetic tree reconstruction

Host spp. code	GenBank Accession & Version No.	Sequenced Species Name
AntPlan	JX524601.1	*Antennaria plantaginifolia*
AstNov	JQ360398.1	*Symphyotrichum novae‐angliae*
BolAst	AF046975.1	*Boltonia asteroides*
CacAtri	KJ418356.1	*Arnoglossum atriplicifolium*
CacPlan	KJ418354.1	*Arnoglossum plantagineum*
CirDis	KC603916.1	*Cirsium discolor*
CorTrip	KM347936.1	*Coreopsis tripteris*
EchPur	GQ864125.1	*Tithonia calva* (contribal)
EupPer	DQ415741.1	*Eupatorium perfoliatum*
EupRug	JQ737035.1	*Ageratina wrightii*
HeleAut	KF607068.1	*Helenium autumnale*
HelHel	AF374914.1	*Trichocoryne connate* (contribal)
HieCan	KT249913.1	*Hieracium umbellatum* (congeneric)
LobCard	AY350630.1	*Lobelia cardinalis*
ParInt	AY947417.1	*Parthenium hysterophorus* (congeneric)
RudHir	AF047901.1	*Helianthus niveus* (contribal)
SilPer	AY196733.1	*Silphium gracile* (congeneric)
VerFas	EF155816.1	*Vernonia fasciculata*
VerMis	KC603926.1|	*Vernonia missurica*

Note

For simplicity, host species names are shortened to the first three letters of the genus, followed by the first 3–4 letters of the species name. The host species codes appear as in Table 1.
